# Large-Scale Shape Transformations of a Sphere Made of a Magnetoactive Elastomer

**DOI:** 10.3390/polym12122933

**Published:** 2020-12-08

**Authors:** Oleg Stolbov, Yuriy Raikher

**Affiliations:** Laboratory of Physics and Mechanics of Soft Matter, Institute of Continuous Media Mechanics, Ural Branch, Russian Academy of Sciences, 614068 Perm, Russia; raikher@icmm.ru

**Keywords:** magnetic polymers, magnetoactive composites, magnetomechanical hysteresis, computer simulation

## Abstract

Magnetostriction effect, i.e., deformation under the action of a uniform applied field, is analyzed to detail for a spherical sample of a magnetoactive elastomer (MAE). A close analogy with the field-induced elongation of spherical ferrofluid droplets implies that similar characteristic effects viz. hysteresis stretching and transfiguration into a distinctively nonellipsoidal bodies, should be inherent to MAE objects as well. The absence until now of such studies seems to be due to very unfavorable conclusions which follow from the theoretical estimates, all of which are based on the assumption that a deformed sphere always retains the geometry of ellipsoid of revolution just changing its aspect ratio under field. Building up an adequate numerical modelling tool, we show that the ‘ellipsoidal’ approximation is misleading beginning right from the case of infinitesimal field strengths and strain increments. The results obtained show that the above-mentioned magnetodeformational effect should distinctively manifest itself in the objects made of quite ordinary MAEs, e.g., composites on the base of silicone cautchouc filled with micron-size carbonyl iron powder.

## 1. Introduction

### 1.1. Field-Induced Striction in Magnetoactive Elastomers

*Magnetoactive elastomer* (MAE) by now has become—successfully consolidating a variety of a number of other definitions—a conventional term to designate a family of soft materials which comprise weakly-linked polymer matrices filled with ferromagnet/ferrite micron-size particles. Due to their macroscopically significant shape and force response to applied magnetic fields and, reciprocally, considerable change of magnetic properties under mechanical loads, MAEs display a diversity of unique magnetomechanical effects. Recent fascinating examples of MAEs use as active elements in micron-scale technical device and medical engineering—field-tuned acoustic metamaterials [1], microfluidic transportation systems [2,3], remotely controlled grippers and microrobots [4,5,6,7]—make it utterly important to fundamentally understand the physics and mechanics underlying the functional properties of these composites.

One of the essential and well known features inherent to those composite materials is their *magnetostriction*, i.e., the effect where a sample changes its shape when subjected to a uniform magnetic field. It is important to distinguish the magnetostriction of MAEs from its namesake term that is used in solid-state magnetism since long ago. The point is that in MAEs the origin of the effect is not the rearrangement of interatomic distances inside the crystal lattice but the process that evolves at a much greater scale in order to minimize the magnetostatic energy of a MAE as an assembly of magnetic particles embedded in an elastic matrix. Due to its entirely different physics, magnetostriction of MAEs is many orders of magnitude greater than that in magnetic crystals and is easily observed in macroscopic experiments. In terms of continuum media electrodynamics, the governing tendency that drives the magnetosctiction of MAEs is a strive to reduce the internal demagnetizing field by way of reshaping of the deformable sample, in which process substantial portions of the material move with respect to each other. Provided that spatial structure of the particles inside each macroscopically small (but containing many micron-size magnets) element remains the same (e.g., homogeneously random) this type of magnetostriction is perfectly prone to be modelled in the framework of a continuum theory.

Along with the macroscopic striction mechanism, in MAEs there exists another response mode which implies particle rearrangements (e.g., aggregation) on the local (mesoscopic) scale. Those structure changes are also governed by the tendency to energy minimization [8,9] and, thus, may collectively contribute to macroscopic deformation [10,11]. The processes of that kind are described with the aid of mesoscopic models [12,13,14]. Given that in standard magnetomechanical experiments there is no possibility to reliably separate the effects of macroscopic and mesoscopic strictions, the issue of their interplay in MAEs is still disputable [10,11,12,13,14]. However, in general, one may be sure that the basics of the magnetostriction effect in MAEs is well established experimentally [15,16,17,18,19,20,21,22] and to a good extent understood theoretically [21,22,23,24,25,26,27,28,29]. Note that the above-presented bibliography is rather incomplete, from a waste array of literature on physics and mechanics of MAEs we have chosen only the works which have the closest relation to the subject. Namely, in below we focus only on the ‘classical’ MAEs, i.e., the systems filled with magnetically soft (low coercive) particles whose spatial arrangement in the matrix is homogeneous and random.

The issue that we analyze here, is a large-scale deformation (morphing) of a spherical sample of MAE induced by magnetostriction. We show that this effect is accompanied by strong shape changes of the initially spherical sample and, under appropriate (and attainable) conditions, acquires hysteresis character. To the best of our knowledge, this interesting and potentially useful phenomenon has never ever been observed experimentally or modelled theoretically with any reasonable accuracy. The major cause for that, in our view, is a gravely unfavorable forecast, once obtained on the basis of seemingly reasonable estimates which turn out to be, although correct qualitatively, but completely misleading quantitatively. In below we, first, present the initial consideration, see Section 2, and then in the following sections show that in quantitative aspect the predicted result differs from a correct one by more than an order of magnitude. Our final results prove that the shape hysteresis of a MAE sample (a sphere is a test body), or at least the pretrasitional regime, might be observed using quite ordinary MAE composites.

### 1.2. MAE Objects and Dense Ferrofluid Droplets: Resemblances and Differences

We treat the problem in the framework of a continuum model since in magnetostriction experiments on conventional MAEs, a sample (sphere, ellipsoid, cylinder, prism, etc.) always elongates in the direction of the field thus evidencing that the reduction of demagnetizing field is the leading effect by far exceeding the mesoscopic contributions. Upon adopting this approach and looking for analogues, one runs into striking similarities between magnetostriction of MAEs and deformation patterns of high-density ferrofluid (FF) drops emerging in result of phase separation in poorly stabilized ferrocolloids. In such droplets the particle concentration is quite large and steric restriction mostly suppresses the mesoscopic rearrangements. On the other hand, the droplet as itself is easily deformable and responds to an applied field with large overall deformations. Moreover, in both cases a body that has elongated under the action of the field, restores its shape when the field is turned off.

For a FF micron-size droplet, a complicated scenario of the field-induced deformation had been discovered yet in 1980’s [30,31,32,33]. Namely, under an increase of the field strength, a spherical droplet, first, stretches just slightly but then, in a threshold-like manner, undergoes a jump-like elongation becoming a distinctively anisometric object in the direction of the field. Moreover, whereas before and just after the abrupt stretch the droplet shape in general resembles a spheroid, upon further increase of the field the droplet transforms to a spindle-like body with distinctively tapered tips. It had been found that: (i) this sequence of states takes place only for the droplets with sufficiently high magnetic permeability and (ii) the occurring deformation is of hysteresis type: upon the field decrease, the droplet shrinks back to a small-eccentricity spheroid at a field strength that is lower than the threshold necessary to induce its elongation [34,35,36].

The particular differences between FF droplets and MAE bodies are clear as well. First, the magnetism of a ferrofluid is due to ferrite (e.g., magnetite) nanoparticles whereas the magnetic sensitivity of a standard MAE is ensured by the presence of micron size particles of a low-coercive ferromagnet (carbonyl iron). This implies that the magnetic susceptibility of a typical MAEs is much greater. Another feature difference is that the integrity of a FF droplet is due to the interface tension that acts at the border of the droplet with the liquid it is floating in. In case of a MAE, the sample integrity is granted by its polymerized body whereas surface tension plays minor rôle. Accordingly, the forces, which restore the initial shape of the object after the field turn-off, have different origin: surficial for droplets and bulk for MAE species.

The internal mechanics of the objects is substantially different as well: whereas inside a fluid content of a FF droplet only isotropic intrinsic stresses might exist, the deformation state of a MAE body is a result of joint action of isotropic (pressure) and shear stresses.

Finally, comparing the prospects of obtaining samples with the size convenient for measurements or applications, one finds out a definite advantage: the MAE spheres may be manufactured with any diameter ranging from millimeters to centimeters, meanwhile the size of FF droplets cannot exceed 20 μm, so that their transformations are observed only under microscope. In other words, there is a strong *a priori* evidence in favor of occurrence of a scale-invariant hysteresis behavior of MAE samples in the magnetostriction regime.

## 2. MAE Sphere under a Uniform Field. Qualitative Analysis

Here we present a a simple comprehensible description of deformation of a MAE sphere, very similar to that used for MAEs in the first magnetostriction studies. Let a sphere of radius *R* be made of a deformable magnetizable incompressible elastic medium. We assume, as it is done in almost all the theoretical papers on droplets and MAEs, that in the course of field-induced elongation the sphere transforms into a prolate spheroid with its major axis pointing along the field direction. Note that, as already mentioned, the ‘spheroid’ hypothesis is not very accurate, it is employed only because of its convenience for qualitative analysis.

Under a uniform field ${\vec{H}}_{0}$, the internal field $\vec{H}$ inside the spheroid is also uniform and coaligned with ${\vec{H}}_{0}$; due to isotropy of the MAE magnetic susceptibility χ, this applies for magnetization $\vec{M}$ as well. Setting the Oz axis of coordinate framework along this direction, one sees that only *z*-projections of all the magnetic vectors are relevant for the problem.

The internal field inside a spheroid in the direction of ${\vec{H}}_{0}$ is

(1) $$H=H_{0}-4\pi NM,$$

where N is the component of demagnetizing tensor in the direction of the field, it is the smaller the more elongated the spheroid. For a prolate spheroid with semi-axes *a* (major) and *b* (minor) and fixed volume $V=\left(4\pi /3\right)R^{3}$, the demagnetizing coefficient N(λ) is a well-known function, see [37], for example, that monotonically tends to zero with the increase of stretch ratio λ=a/R.

Then the magnetic energy of the model MAE body derived from the general expression [37] is

(2) $$E_{\mathrm{mag}}=E_{\mathrm{mag}}V,\;E_{\mathrm{mag}}=-\int_{0}^{H_{0}}M\left(H,N(\lambda )\right)dH_{0},$$

where $E_{\mathrm{mag}}$ is the magnetic energy density.

The assumption of spheroidal shape of the deformed sphere entails that the acquired deformation is homogeneous. Then, describing the excess of the elastic energy under strain λ by the two-parameter Peng–Landel model [38] one gets

(3) $$E_{\mathrm{el}}=E_{\mathrm{el}}V,\;E_{\mathrm{el}}=\frac{1}{2}G\left(J^{-2/3}\left(\lambda^{2}+\frac{2}{\lambda }\right)-3\right)+\frac{1}{2}K{\left(J-1\right)}^{2}\;\;\underset{J=1}{\Rightarrow }\;\frac{1}{2}G\left(\lambda^{2}+\frac{2}{\lambda }-3\right);$$

here $E_{\mathrm{el}}$ is the elastic energy density, *J* the determinant of deformation gradient tensor, whereas *G* and *K* are the shear and bulk moduli, respectively. For an incompressible material (J=1) the elastic potential transforms to the neo-Hookean one, as indicated by the last part of Equation (3). At small perturbations (λ−1)≪1 Formula (3) reduces to a simple Hooke law.

Uniting expressions (2) and (3), one arrives at the magnetoelastic energy density $E_{m+e}=E_{\mathrm{mag}}+E_{\mathrm{el}}$ of a model MAE body under field $H_{0}$. Equilibrium configurations of the spheroid are determined from minimization of function $E_{\mathrm{me}}$ with respect to λ at a given $H_{0}$. Differentiation with allowance for Equation (1), yields the energy extremum equation $\partial E_{m+e}/\partial \lambda =0$ in the form

(4) $$G\lambda \left(1-\lambda^{-3}\right)+\pi M^{2}\;\frac{\partial N}{\partial \lambda }=0.$$

We consider Equation (4) for a linear magnetization law M=χH, where magnetic susceptibility χ is constant all over the sample. Even under those facilitating assumptions, an explicit analytic form is available not for the magnetodeformational curve $\lambda (H_{0})$ but for the inverse function that is

(5) $$H_{0}\left(\lambda \right)=\left[\frac{1}{\chi }+4\pi N\left(\lambda \right)\right]\sqrt{-\frac{G\lambda (1-1/\lambda^{3})}{\pi \;\partial N(\lambda )/\partial \lambda }}\;\;\mathrm{or}\;\;\frac{H_{0}}{\sqrt{G}}=\left[\frac{1}{\chi }+4\pi N\left(\lambda \right)\right]\sqrt{-\frac{\lambda (1-1/\lambda^{3})}{\pi \;\partial N(\lambda )/\partial \lambda }}$$

where the last expression is nondimensional.

**Figure 1:**
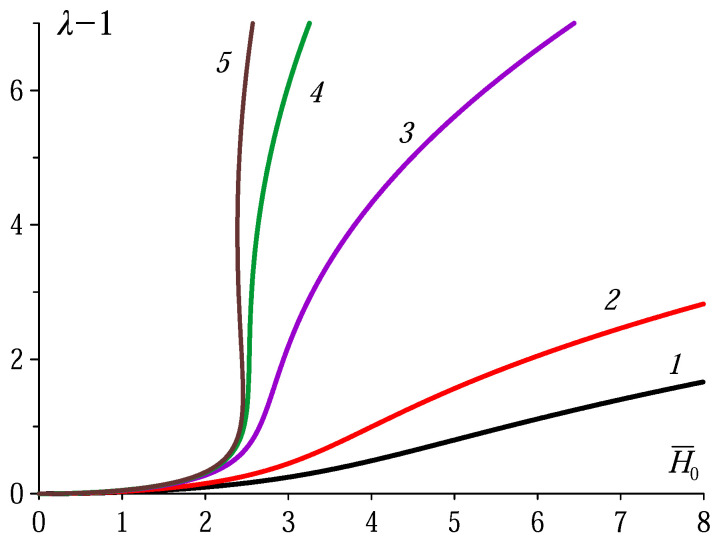
Magnetodeformational curves for a sphere made of a linearly magnetizable MAE with neo-Hookean stress-strain law. The curves (right to left) correspond to the values of magnetic susceptibility χ [in CGS units]: 0.5 (1), 1.0 (2), 6.0 (3), 20 (4) and 40 (5); the external field strength is scaled as ${\bar{H}}_{0}=H_{0}/\sqrt{G}$.

Figure 1 shows magnetodeformational curves—the increment of nondimensional stretch ratio λ−1 as a function of nondimensional external field ${\bar{H}}_{0}$—rendered by Equation (5). As it is seen, at sufficiently high χ the curves, instead of being single-valued, become S-shaped, so that one and the same field strength (imagine a vertical line at ${\bar{H}}_{0}$ slightly above 2) may correspond to either of two stable MAE spheroids, whose stretch ratios are notably different. Therefore, this schematic consideration predicts that a MAE sphere, provided its material parameters are appropriate, responds to applied uniform field in a hysteresis way: at increasing field, first, becomes a slightly elongated spheroid but further on enhances its anisometricity (eccentricity) in a jump-like way.

From the curves of Figure 1 it follows that the curves acquire the S-shaped profile as soon as χ exceeds the value delivered by the root $\chi_{*}$ of equation

(6) $$dH_{0}\left(\chi ,\lambda \right)/d\lambda =0.$$

Solution of Equation (6) yields $\chi_{*}≃25$ [in CGS units], so that the corresponding SI susceptibility is an order of magnitude higher. Given the magnetic parameters of real MAEs, one concludes that neither composite of that kind can even approach the requirement. In this connection, we point out Ref. [39] where the same, as above, problem was solved for the Mooney-Rivlin elastic potential of the matrix, and had rendered very much similar results. In particular, the authors have obtained analytically the threshold value of magnetic susceptibility of a MAE as $\chi_{*}≃23$ [CGS units] that is virtually the same as $\chi_{*}≃25$ that we have evaluated numerically.

It is important to remark that the ‘spheroidal’ hypothesis worked very well for FF droplets in qualitative as well as in quantitative aspect [32,33]. This implicitly makes it to seem well reliable with respect to MAE spheres, and that—in view of the above-obtained estimates—entirely excludes a chance to observe a hysteresis stretching of a MAE sphere.

The reason that makes one to doubt good applicability of ‘spheroid’ approximation in the MAE case turns up when one recalls the fundamental difference in mechanical stress distributions in fluid and elastic objects. Namely, this is the presence of shear stresses in elastic bodies; in formal terms—The non-diagonality of the internal stress tensor. The crucial rôle of the latter had been demonstrated in 2005 by Raikher and Stolbov [24] who had found that neither magnetic nor mechanical stress fields may be considered to be uniform yet under infinitesimal nonsphericity of an initially spherical MAE body. Although those shape deviations are hardly discernible in visually presentations, the energy gain due to spatial non-uniformities of both fields ranges tens of percent. With allowance for that, and taking into account that a sphere with χ in the ‘pretransitional’ range ($\chi ∼\chi_{*}$) displays a very steep growth of λ with $H_{0}$ (see curves 4 and 5 in Figure 1), one can justly surmise that the non-ellipticity, once appeared, would drastically enhance in the high-field regime. In particular, this implies that the initially rounded ends of the former sphere would taper and resemble rather cones than smooth convexes. To address these issues, in the following we perform numerical modelling of the problem by means of finite element method.

## 3. MAE Sphere under a Uniform Field: A Coupled Magnetoelastic Problem

### 3.1. Finite Deformations Approach

Anticipating large shape changes, the problem is formulated in terms of finite strains. For that, two configurations are introduced: the initial and actual ones, so that to the radius-vector $\vec{r}$ defined in the initial configuration corresponds the radius-vector $\vec{R}=\vec{r}+\vec{u}$ in the actual configuration, here $\vec{u}$ is displacement vector. The basis vectors are defined as ${\vec{\epsilon }}_{i}=\partial \vec{r}/\partial q_{i}$ and ${\hat{\vec{\epsilon }}}_{i}=\partial \vec{R}/\partial q_{i}$ in the initial and actual configurations, respectively; here $q_{i}$ are generalized coordinates.

Hamilton operators in the initial and actual configurations are introduced as $\nabla ={\vec{\epsilon }}^{\;i}\partial /\partial q_{i}$ and $\hat{\nabla }={\hat{\vec{\epsilon }}}^{\;i}\partial /\partial q_{i}$ where ${\vec{\epsilon }}^{\;i}$ and ${\hat{\vec{\epsilon }}}^{\;i}$ are the vectors of respective reciprocal bases. Introducing fundamental kinematic function—deformation gradient—as

(7) $$F={\left(\nabla \vec{R}\right)}^{T}={\hat{\vec{\epsilon }}}_{i}{\vec{\epsilon }}^{\;i}=g+\nabla {\vec{u}}^{\;T},$$

where g is metric (unit) tensor and index *T* denotes transposition, for the inverse function one has

(8) F−1=(∇2^r→)T=ϵ→iϵ→^i=g−∇u→T.

In these terms, the Hamilton operators in initial and actual configurations are related to each other as

(9) ∇2^=F−1·∇.

A generic expression for an elastic potential of initially isotropic continuum may be presented in the form

(10) $$W=W\left(I_{1}\left(C\right),\;I_{2}\left(C\right),\;I_{3}\left(C\right)\right),$$

where $I_{1}\left(\cdot \right)=\mathrm{Tr}\left(\cdot \right)$, $I_{2}$, $I_{3}=\mathrm{Det}\left(\cdot \right)$ are the main tensor invariants of the right Cauchy–Green deformation tensor C. With potential (10), the Piola-Kirchhoff tensor of second kind $P_{II}=2\partial W/\partial C$ takes the form [40]

$$P_{II}=2\left(\frac{\partial W}{\partial I_{1}}\frac{\partial I_{1}}{\partial C}+\frac{\partial W}{\partial I_{2}}\frac{\partial I_{2}}{\partial C}+\frac{\partial W}{\partial I_{3}}\frac{\partial I_{3}}{\partial C}\right)$$

and the Cauchy stress tensor writes

(11) $$T=J^{-1}F\;\cdot \;P_{II}\;\cdot \;F^{T}$$

with $J=I_{3}\left(F\right)$ being the Jacobian of deformation gradient tensor.

### 3.2. Elasticity Energy

To describe the mechanical behavior of an MAE in a realistic way, we choose *W* in the Peng-Landel form [38] since it is known to be well appropriate for slightly compressible elastomers at large strains:

(12) $$W=\frac{1}{2}G\left(J^{-2/3}I_{1}\left(C\right)-3\right)+\frac{1}{2}K{\left(J-1\right)}^{2};$$

we set K=500G.

To solve the problem in actual configuration, the space of entire calculation box Ω is split in two parts: $\Omega_{\mathrm{sam}}$ that is the sample and $\Omega_{\mathrm{sur}}$ that is its surrounding. To maintain continuity of the deformation gradient F everywhere in Ω, we ascribe to the sample surrounding an elasticity potential in the same form as (12) but with modulus $G_{s}$ that is several orders of magnitude lower than *G* to make a particular value of $G_{s}$ irrelevant for final results. Under those conditions, the elastic energy of the system is

(13) $$U_{\mathrm{el}}=\int_{\Omega_{\mathrm{sam}}}WdV_{0}+k_{s}\int_{\Omega_{\mathrm{sur}}}WdV_{0},$$

where $k_{s}=G_{s}/G=10^{-5}$ and $dV_{0}=dV/J$ is the volume element in the initial configuration.

### 3.3. Magnetic Energy

In the absence of electric currents—we assume that conductivity of MAE is negligible—The magnetic field $\vec{H}$ inside the system might be presented as a gradient of a scalar function ψ in the actual configuration:

(14) H→=H→0−∇2^ψ

, where ${\vec{H}}_{0}$ denotes external field.

Expression for the magnetic energy density increment valid for any point of the space Ω is [37]:

(15) $$\delta W_{\mathrm{mag}}=-\frac{1}{4\pi }\vec{B}\;\cdot \;\delta \vec{H},$$

with $\vec{B}=\vec{H}+4\pi \vec{M}$ being magnetic induction vector that incorporates field $\vec{H}$ and magnetization vector $\vec{M}$.

Under assumption of linear magnetization law $\vec{M}=\chi \vec{H}$ with a constant isotropic susceptibility χ, the magnetic energy density is

(16) $$W_{\mathrm{mag}}=-\frac{1}{8\pi }\left(1+4\pi \chi \right)H^{2},$$

so that the magnetic part of the system energy takes the form

(17) $$U_{\mathrm{mag}}=\int_{\Omega }W_{\mathrm{mag}}J\;dV_{0}=-\frac{1}{8\pi }\int_{\Omega }H^{2}J\;dV_{0}-\frac{1}{2}\chi \int_{\Omega_{\mathrm{sam}}}H^{2}J\;dV_{0},$$

where the field $\vec{H}$ (14) is transformed to the actual configuration with the aid of Hamilton operator as

(18) $$\vec{H}={\vec{H}}_{0}-F^{-1}\;\cdot \;\nabla \psi .$$

Uniting Equations (13) and (17), one arrives at the expression for the joint magnetoelastic energy of the system:

(19) $$U\left(\nabla \vec{u},\nabla \psi \right)=-\frac{1}{8\pi }\int_{\Omega }H^{2}J\;dV_{0}+k_{s}\int_{\Omega_{sur}}W\;dV_{0}+\int_{\Omega_{\mathrm{sam}}}\left(W-\frac{1}{2}J\chi H^{2}\right)\;dV_{0},$$

which minimum determines the equilibrium shape of the MAE sample under a given field. For the problem under study, the general form of variational equation to be solved is

(20) $$\delta U=\frac{\partial U}{\partial (\nabla \vec{u})}\;\cdot \cdot {\left(\nabla \delta \vec{u}\right)}^{T}+\frac{\partial U}{\nabla \psi }\;\cdot \;\nabla \delta \psi =0.$$

## 4. Method of Solution

The problem has evident axial symmetry around the direction of applied field. Accordingly, a cylindrical coordinate frame (ρ,z,φ) is introduced with polar axis along ${\vec{H}}_{0}$. Making further use of the symmetry, we consider only a space region abutting the quarter (1st quadrant) of the circle of radius 10R that is perpendicular to Oz and centered at the coordinate origin, the outer border of this region is denoted as Γ.

Equation (20) is solved numerically by the finite-element numerical method realized in the algorithms of FEniCS computing platform [41,42]. The built up mesh is nonuniform, it is most dense at the central part of Ω and gradually becomes more sparse when approaching Γ. Two functions: $\vec{u}\left(\rho ,z\right)$ and ψ(ρ,z) defined in a mixed finite-element space $\left(P_{3},P_{1}\right)$ inside Ω, are evaluated under boundary conditions

(21) $$\;u_{\rho }{|}_{\rho =0}=0,\;u_{z}{|}_{z=0}=0;\;\psi =0\;\;\mathrm{on}\;\;\Gamma .$$

The strength of applied field ${\vec{H}}_{0}$ is varied gradually in small steps. Equation (20) is solved anew for each value of $H_{0}$ with boundary conditions (21) on the adopted mesh by Newton method (implemented in FEniCS platform); the values of $\vec{u}\left(\rho ,z\right)$ and ψ(ρ,z) obtained in result of a given calculation step are taken as initial ones when commencing the next step. For the case of finite deformations, the stretch ratio is evaluated according to $\lambda =1+u_{z}\left(\rho =0,\;z=R\right)/R$ that fully complies with the previous definition since for a spheroidal case $R+u_{z}\left(\rho =0,\;z=R\right)=a$.

## 5. Results and Discussion

A simple test for correctness of the obtained results follows from comparison with the dependence derived analytically in the framework of perturbation theory in Ref. [24]; when recalculated for a linearly magnetizable MAE it yields

(22) $$\lambda -1=\frac{20\chi^{2}{\bar{H}}_{0}^{2}}{57{\left(1+\frac{4\pi }{3}\chi \right)}^{2}}.$$

As already mentioned, formula (22) had been obtained beyond the ‘spheroidal’ approximation: the magnetic and mechanical stress fields inside the MAE sphere are nonuniform.

**Figure 2:**
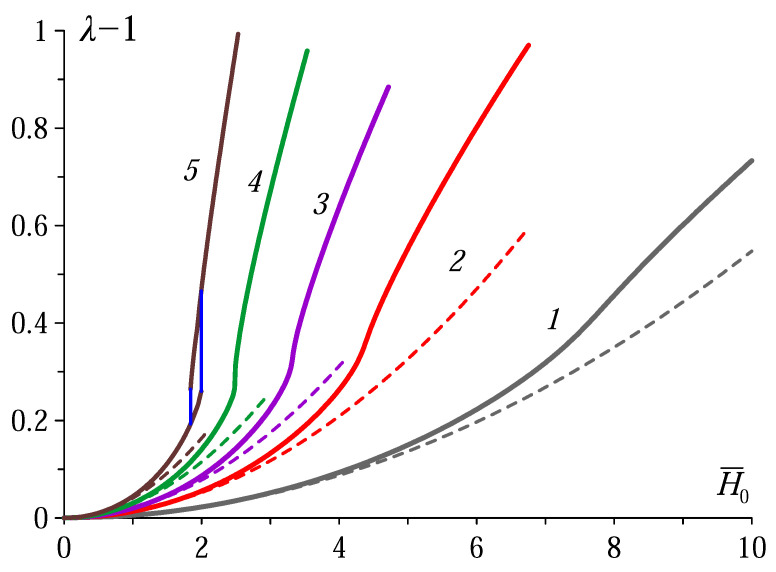
Magnetodeformational curves for a sphere made of a linearly magnetizable MAE with Peng–Landel stress-strain law. Solid curves are obtained by numerical modelling, dashed curves render dependence (22). The curves (right to left) correspond to the values of magnetic susceptibility χ [in CGS units]: 0.1 1), 0.2 (2), 0.3 (3), 0.5 (4) and 1.0 (5); the external field strength is nondimensionalized as ${\bar{H}}_{0}=H_{0}/\sqrt{G}$.

The results of numerical modelling, which scheme is outlined in Section 3 and Section 4, are shown in Figure 2 together with plots of the perturbation-approach formula (22).

As seen, quite expectably, the perturbation theory works the better the lower the susceptibility. A much more striking feature is that, in comparison with the parameters of the plots of Figure 1, the values of χ which ensure strong elongation, are 40–50 times lower than those predicted by the ‘spheroidal’ approximation. This proves that prognostic ability of the latter in the MAE case is very limited. Apparently, there is only general qualitative likeness between the plots of Figure 1 and Figure 2 and, this completely changes the view on the possibility to really access the effect. For example, according to Figure 2 a well discernible hysteresis loop emerges at χ>0.5, see curve *5* that corresponds to χ=1. Certainly, even with the so much reduced estimate, to find a MAE with χ>0.5 among now available systems is hardly possible. However, even at χ∼0.2 the growth of $\lambda ({\bar{H}}_{0})$ is much faster than parabolic, and this is an unambiguous signature of the pretransition regime. To test such a behavior should not be difficult since χ∼0.20–0.24 is a typical value for standard MAEs filled with carbonyl iron under the particle volume fraction around 30% [8,43,44].

**Figure 3:**
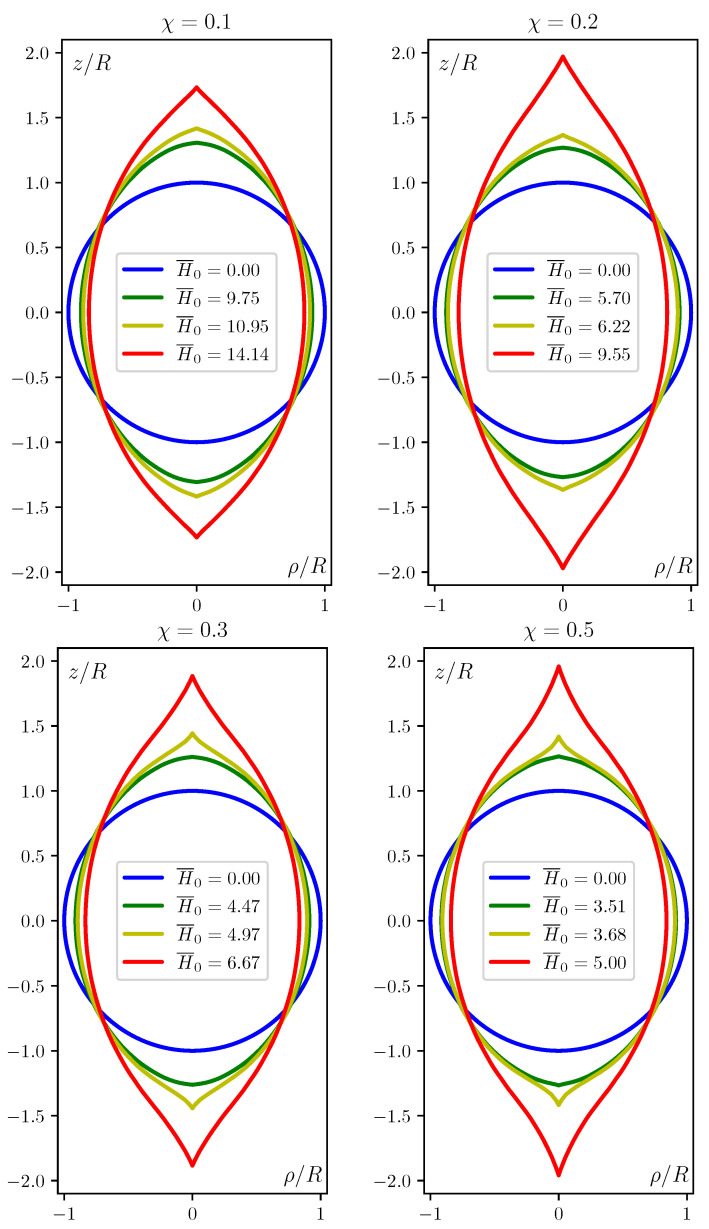
Meridional cross-sections of the shapes assumed by a MAE sphere under increasing field; the field is directed along Oz axis and ρ is the transverse cylindrical coordinate. The magnetic susceptibility χ and field strength ${\bar{H}}_{0}$ values are indicated at each panel.

**Figure 4:**
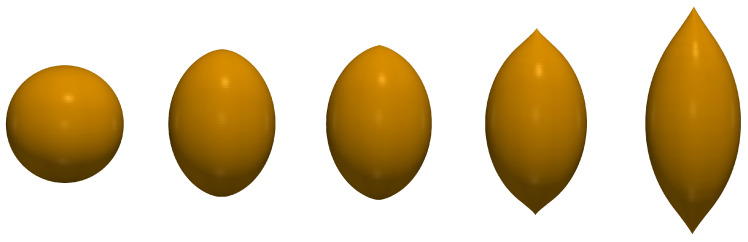
Shape evolution of a MAE sphere with χ=0.2 under increasing magnetic field; the nondimensional field strengths are (from left to right): ${\bar{H}}_{0}=0$; 5.7; 6.12; 7.42 and 9.55.

Another and unavoidable drawback of the ‘spheroidal’ hypothesis comes out when the overall shapes assumed by a magnetized MAE sphere are investigated with the aid of correct numerical modelling. The sequences of the obtained configurations are shown in Figure 3 and Figure 4. Notably, independently of actual value of χ all the high-field shapes acquire tapered tips where the local geometry is completely different. Namely, one of the two main surface indices changes sign: the meridional curvature, being positive (convex) at the main part of the body, becomes negative (concave) at the lateral surface of the tips.

For completeness and in connection with the magnetomechanical hysteresis phenomena in MAEs, we remark that the above-discussed situation is not the only case where such a scenario is encountered. In particular, the shape transition of a distinctive hysteresis type was discovered experimentally by Zrínyi et al. [45], the theoretical explanation was given in Refs. [39,45]. A rod-like sample made of a very soft MAE (ferrogel), was positioned in such a way that one of its ends was close to a field source (solenoid). On increasing the current in the solenoid, the rod underwent an abrupt elongation in the direction of the field source, whereas on the current turn-off it restored its initial state passing, on its way back, the stage of jump-like shrinking. The intensity of electric current under which the jump-like stretch occurred was considerably higher than that at which the rod restored its initial length. This interesting example of hysteretic behavior of a MAE is, however, essentially different from the magnetostriction situation that we study here. Namely, the stimulus that compels the rod to deform originates from a nonuniform magnetic field, so that there is a non-zero force exerted on its center of mass. Meanwhile, in our problem, the MAE object is free-standing as in a uniform field there is no net force acting on it.

## 6. Conclusions

- Theoretical evidence is presented that magnetostriction effect in free-standing MAE samples—sphere is a test object—could be understood and studied adequately only with the aid of a detailed magnetomechanical description. The basic cause for that is that only the finite-element or alike methods are able to account for the virtually infinite number of degrees of freedom of a deformable elastic body.
- As soon as a powerful numerical tool is applied, it turns out that the estimates obtained with ‘spheroidal’ approximation could be used exclusively for qualitative analysis and have no quantitative validity. The here obtained solution refutes the former prediction of virtual impossibility to observe magnetomechanical hysteresis of MAE samples and moves the appropriate parameter interval to a real range.
- Even if the magnetic susceptibility of a MAE is not high enough to ensure a real jump of the stretch ratio λ, function $\lambda (H_{0})$ signals on the proximity of the hysteresis regime by a characteristic inflexion. Besides that, and contrary to the case of ferrofluid droplets, in MAE objects the effect has no size limitations and may well occur at macroscopic scale.
- The model that we use is adequate for the problem solved but is limited to statics and linearly magnetiable MAEs. A first step forward, utterly necessary and not extremely laborious, should be its extension for a nonlinear magnetization law since magnetic saturation is a fundamental feature of MAEs; such an improvement would bring theoretical predictions closer to real situation.
- In our view, for further advances the model should be developed along the following lines:
  – it should allow for re-distribution of the filler particles, which, albeit elastically impeded by the MAE matrix, possess some translational freedom that is the greater the softer the elastomer. Due to geometry reasons, the internal field gradients in the tips exceed those in the middle section of the body, so that the magnetic forces urge the particles to the tips. The augmented particle concentration enhances the local magnetic susceptibility of the tip, and this, in turn, affects its geometry. At present, the net effect of this interplay is unknown.
  – it should be extended to have a dynamic formulation. This would give an opportunity to estimate the response time of a MAE sphere shape morphing that, obviously, would strongly depend on the object size. Besides that, a large number of cases where MAE objects of various shapes may undergo field-controlled motion and locomotion would become accessible for reliable predcitions. Evidently, this class of problems is very interesting from a great many of applicational viewpoints.

## References

[1] Yu K., Fang N.X., Huang G., Wang Q. (2018). Magnetoactive acoustic metamaterials. Adv. Mater..

[2] Behrooz M., Gordaninejad F. (2016). A flexible microfluid transport system featuring magnetorheological elastomer. Smart Mater. Struct..

[3] Yuan Y., Yalikun Y., Ota N., Tanaka Y. (2018). Property investigation of replaceable PDMS membrane as an actuator in microfluidic device. Actuators.

[4] Hu W., Lum G.Z., Mastrangeli M., Sitti M. (2018). Small-scale soft-bodied robot with multimodal locomotion. Nature.

[5] Alekhina Y.A., Makarova L.A., Kostrov S.A., Stepanov G.V., Kazimirova E.G., Perov N.S., Kramarenko E.Y. (2019). Development of magnetoactive elastomers for sealing eye retina detachments. J. Appl. Polym. Sci..

[6] Alapan Y., Karacakol A.C., Guzelhan S.N., Isik I., Sitti M. (2020). Reprogrammable shape morphing of magnetic soft machines. Sci. Adv..

[7] Zhalmuratova D., Chung H.-J. (2020). Reinforced gels and elastomers for biomedical and soft robotics applications. ACS Appl. Polym. Mater..

[8] Stepanov G.V., Borin D.Y., Raikher Y.L., Melenev P.V., Perov N.S. (2008). Motion of ferroparticles inside the polymeric matrix in magnetoactive elastomers. J. Phys. Condens. Matter.

[9] Biller A.M., Stolbov O.V., Raikher Y.L. (2014). Modeling of particle interactions in magnetorheological elastomers. J. Appl. Phys..

[10] Stolbov O.V., Raikher Y.L., Balasoiu M. (2011). Modelling of magnetodipolar striction in soft magnetic elastomers. Soft Matter.

[11] Gong X., Liao G., Xuan S. (2012). Full-field deformation of magnetorheological elastomer under uniform magnetic field. Appl. Phys. Lett..

[12] Menzel A. (2014). Bridging from particle to macroscopic scales in uniaxial magnetic gels. J. Chem. Phys..

[13] Pessot G., Weeber R., Holm C., Löwen H., Menzel A. (2015). Towards a scale-bridging description of ferrogels. J. Phys. Condens. Matter.

[14] Fischer L., Menzel A. (2019). Magnetostriction in magnetic gels and elastomers as a function of the internal structure and particle distribution. J. Chem. Phys..

[15] Ginder J.M., Clark S.M., Schlotter W.F., Nichols M.E. (2002). Magnetostrictive phenomena in magnetorheological elastomers. Int. J. Mod. Phys. B.

[16] Gollwitzer C., Turanov A., Krekhova M., Lattermann G., Rehberg I., Richter R. (2008). Measuring the deformation of a ferrogel sphere in a homogeneous magnetic field. J. Chem. Phys..

[17] Guan X., Dong X., Ou J. (2008). Magnetostrictive effect of magnetorheological elastomer. J. Magn. Magn. Mater..

[18] Filipcsei G., Zrínyi M. (2010). Magnetodeformation effects and the swelling of ferrogels in a uniform magnetic field. J. Phys. Condens. Matter.

[19] Diguet G., Beaugnon E., Cavaillé J.Y. (2010). Shape effect in the magnetostriction of ferromagnetic composite. J. Magn. Magn. Mater..

[20] Andriuschenko P., Nefedev K., Stepanov G. (2014). Calculations of magnetoactive elastomer reactions in a uniform external magnetic field. Eur. Phys. J. B.

[21] Saveliev D.V., Belyaeva I.A., Chashin D.V., Fetisov L.Y., Romeis D., Kettl W., Kramarenko E.Y., Saphiannikova M., Stepanov G.V., Shamonin M. (2020). Giant extensional strain of magnetoactive elastomeric cylinders in uniform magnetic fields. Materials.

[22] Romeis D., Kostrov S.A., Kramarenko E.Y., Stepanov G.V., Shamonin M., Saphiannikova M. (2020). Magnetic-field-induced stress in confined magnetoactive elastomers. Soft Matter.

[23] Raikher Y.L., Stolbov O.V. (2000). Magnetodeformational effect in a ferroelastic material. Tech. Phys. Lett..

[24] Raikher Y.L., Stolbov O.V. (2005). Deformation of an ellipsoidal ferrogel sample in a uniform magnetic field. J. Appl. Mech. Tech. Phys..

[25] Raikher Y.L., Stolbov O.V. (2008). Numerical modeling of large field-induced strains in ferroelastic bodies: A continuum approach. J. Phys. Condens. Matter.

[26] Morozov K., Shliomis M., Yamaguchi H. (2009). Magnetic deformation of ferrogel bodies: Procrustes effect. Phys. Rev. E.

[27] Ivaneyko D., Toshchevikov V., Saphiannikova M., Heinrich G. (2014). Mechanical properties of magneto-sensitive elastomers: Unification of the continuum-mechanics and microscopic theoretical approaches. Soft Matter.

[28] Zubarev A.Y., Borin D.Y. (2015). Effect of particle concentration on ferrogel magnetodeformation. J. Magn. Magn. Mater..

[29] Romeis D., Metsch P., Kästner M., Saphiannikova M. (2017). Modeling and simulation of magnetorheological elastomers: A comparison of continuum and dipole approaches. Phys. Rev. E.

[30] Arkhipenko V.I., Barkov Y.D., Bashtovoi V.G. (1978). Study of a magnetized fluid drop shape in a homogeneous magnetic field. Magnetohydrodynamics.

[31] Drozdova V.I., Skrobotova T.V., Chekanov V.V. (1979). Experimental study of the hydrostatics of the interphase surface of a ferrofluid. Magnetohydrodynamics.

[32] Bacri J.-C., Salin D., Massart R. (1982). Study of the deformation of ferrofluid droplets in a magnetic field. J. Phys. Lett..

[33] Bacri J.-C., Salin D. (1982). Instability of ferrofluid magnetic drops under magnetic field. J. Phys. Lett..

[34] Cebers A. (1985). Virial method of investigation of statics and dynamics of magnetizable liquids. Magnetohydrodynamics.

[35] Afkhami S., Tyler A.J., Renardy Y., Renardy M., St. Pierre T.G., Woodward R.C., Riffle J.S. (2010). Deformation of a hydrophobic ferrofluid droplet suspended in a viscous medium under uniform magnetic fields. J. Fluid Mech..

[36] Misra K. (2020). Magnetic (electric) drop deformation in uniform external fields: Volume averaged methods and formation of static and dynamic conical tips. Phys. Fluids.

[37] Landau L.D., Lifshitz E.M., Pitaevskii L.P. (1984). Electrodynamics of Continuous Media.

[38] Peng S.T.J., Landel R.F. (1975). Stored energy function and compressibility of compressible rubberlike materials under large strain. J. Appl. Phys..

[39] Naletova V.A., Pelevina D.A., Merkulov D.I., Zeidis I., Zimmermann K. (2016). Bi-stability of the deformation of a body with a magnetizable elastomer in a magnetic field. Magnetohydrodynamics.

[40] Lurie A.I. (1990). Nonlinear Theory of Elasticity.

[41] Alnæs M.S., Blechta J., Hake J., Johansson A., Kehlet B., Logg A., Richardson C., Ring J., Rognes M.E., Wells G.N. (2015). The FEniCS project version 1.5. Arch. Numer. Softw..

[42] FEniCS Project. https://fenicsproject.org.

[43] Kashima S., Myasaka F., Hirata K. (2012). Novel soft actuator using magnetorheological elastomer. IEEE Trans. Magn..

[44] Mitsumata T., Ohori S., Honds A., Kawai M. (2013). Magnetism and viscoelasticity of magnetic elastomers with wide range modulation of dynamic modulus. Soft Matter.

[45] Zrínyi M., Barsi L., Szabó L., Kilian H.-G. (1997). Direct observation of abrupt shape transition in ferrogels induced by nonuniform magnetic field. J. Chem. Phys..

